# A Comparison of Internal Fixation and Hemiarthroplasty for Nondisplaced Femoral Neck Fractures: An Updated Systematic Review and Meta-Analysis

**DOI:** 10.7759/cureus.105817

**Published:** 2026-03-25

**Authors:** Mohamed Xamza, Eyad Jamileh, Adam Khan, Mohamed S Baalawi, Mohannad Jamileh

**Affiliations:** 1 Orthopedics and Trauma, Barking, Havering, and Redbridge (BHR) NHS Teaching Hospital, London, GBR; 2 Medicine, East Lancashire Hospitals NHS Trust, Blackburn, GBR; 3 Orthopedics and Trauma, Barts and The London School of Medicine and Dentistry, London, GBR; 4 Emergency Medicine, Barts Health NHS Trust, London, GBR; 5 Orthodontics, Barts and The London School of Medicine and Dentistry, London, GBR

**Keywords:** elderly patients, femoral neck fractures, hemiarthroplasty, internal fixation, orthopedics

## Abstract

The optimal management of nondisplaced femoral neck fractures (FNFs) in elderly patients remains debated. Internal fixation (IF) offers shorter operative time and reduced blood loss but carries risks of nonunion and reoperation, whereas hemiarthroplasty (HA) may reduce these failures at the expense of greater operative burden. We conducted a systematic search of PubMed, MEDLINE, and Embase (completed in September 2025) to identify randomized controlled trials comparing IF and HA in patients aged ≥65 years with Garden I-II FNFs. Four RCTs were included, enrolling 454 patients (230 IF; 224 HA). Outcomes included implant-related complications, reoperation, mortality, functional outcomes, perioperative variables, and quality of life. IF was associated with significantly higher implant-related complications (19.1% vs. 3.1%; odds ratio (OR) = 4.12, 95% confidence interval (CI) = 2.12-8.00; P < 0.0001) and reoperations (OR = 4.10, 95% CI = 2.12-7.95; P < 0.0001). Osteonecrosis was more frequent following IF (OR = 5.97, 95% CI = 1.54-23.14; P = 0.01), as was nonunion/fixation failure (OR = 13.43, 95% CI = 3.66-49.33; P < 0.0001). Deep infection rates were comparable between the groups (OR = 0.52, 95% CI = 0.14-1.92; P = 0.33). In contrast, HA was associated with greater blood loss (mean difference (MD) = -143.11 mL, 95% CI = -204.53 to -81.69; P < 0.0001) and longer operative time (MD = -23.07 minutes, 95% CI = -41.14 to -4.99; P = 0.01). Mortality was comparable between the groups up to 36 months (risk ratio = 0.93, 95% CI = 0.50-1.73; P = 0.81). Early functional outcomes favored HA, although long-term scores were similar. This meta-analysis suggests that HA reduces complications and reoperations without increasing mortality and may represent a preferable option for many frail elderly patients, while treatment decisions should remain individualized based on operative risk and comorbidity.

## Introduction and background

Hip fractures are a major health burden in contemporary orthopedic practice, with approximately 70,000 cases reported annually in the United Kingdom over the past five years [1]. Femoral neck fractures (FNFs) represent nearly half of all hip fractures and are associated with considerable morbidity and mortality, with postoperative mortality rates reported as high as 20% [1,2]. Internal fixation (IF) preserves the native femoral head but carries risks such as nonunion and fixation failure, whereas hemiarthroplasty (HA) replaces the femoral head and avoids these complications at the expense of a more invasive procedure, such as avascular necrosis, malunion, infection, and hemorrhage, highlighting the importance of selecting the most appropriate intervention [2,3].

Nondisplaced fractures (Garden I-II) are generally considered stable injuries and are commonly managed with IF, although the optimal treatment approach remains debated for displaced (Garden III-IV) categories [4]. Treatment strategies differ accordingly: arthroplasty has been generally favored for displaced FNFs, whereas the optimal approach for nondisplaced FNFs remains uncertain [5,6]. Despite numerous studies, there is no clear consensus, and management often reflects surgeons' preferences or institutional practices [2,5].

This systematic review and meta-analysis focuses exclusively on nondisplaced FNFs. It aims to synthesize the latest comparative evidence that has emerged between IF and HA, evaluating outcomes such as postoperative complications, infection, pain, and functional recovery. This study seeks to inform evidence-based recommendations and guide clinical decision-making in an area of ongoing debate.

## Review

Methods

This systematic review and meta-analysis was conducted and reported in accordance with the Preferred Reporting Items for Systematic Reviews and Meta-Analyses (PRISMA) 2020 statement [7]. The protocol was prospectively registered in the International Prospective Register of Systematic Reviews (CRD420251138564). The PRISMA 2020 flow diagram was generated using the PRISMA 2020 flow diagram software [8].

Eligibility Criteria

This study aimed to evaluate the comparative outcomes of IF and HA in patients with nondisplaced FNFs by synthesizing available evidence from randomized controlled trials (RCTs).

Studies were included if they directly compared IF and HA. Eligible studies had to report at least one of the primary or secondary outcomes relevant to the review. The intervention group comprised patients who underwent IF, while the comparator group included those treated with HA. Eligibility criteria placed no restrictions on participant demographics such as age, sex, or comorbidities, other than requiring patients to be aged 65 years or older. Only studies published in English were included. For studies with more than two intervention arms, only the relevant comparison groups (IF and HA) were included in the analysis, with noncomparable arms excluded to avoid duplication of data.

Studies were excluded if they were case reports, single-arm observational studies without a comparator, narrative reviews, or conference abstracts. Additionally, studies that did not report the prespecified outcomes were excluded.

Primary Outcomes

The primary outcomes included implant-related complications, as defined by each study, and the individual complication subcategories: nonunion/fixation failure, osteonecrosis, and peri-implant fracture. Mortality at specific time points (one, two, and three years) was also considered a primary endpoint [9-12].

Secondary Outcomes

The secondary outcomes included total complications (both implant-related and general), reoperation rates, perioperative variables such as intraoperative blood loss and operative time, and functional outcomes, including Harris Hip Score [13] and social independence, at reported follow-up intervals.

Literature Search Strategy

A comprehensive literature search was conducted by two independent reviewers across multiple electronic databases, including MEDLINE (via PubMed), EMBASE, and the Cochrane Central Register of Controlled Trials (CENTRAL). The final search was completed on September 4, 2025. The complete search strategies are fully described in the Appendix. In addition to database searches, gray literature, and ongoing or unpublished trials were identified through searches of the World Health Organization International Clinical Trials Registry Platform [14], ClinicalTrials.gov [15], and the International Standard Randomised Controlled Trial Number Register [16]. To ensure thorough coverage, reference lists of all included studies and relevant systematic reviews were manually screened for additional eligible articles.

Study Selection

Two independent reviewers, MX and EJ (blinded to each other’s assessments), screened all retrieved studies for eligibility based on titles and abstracts. Full-text articles were obtained for studies that appeared to meet the inclusion criteria. If discrepancies in the selection process were identified, they were resolved through discussion or consultation with a third reviewer, MSB. The characteristics of the studies are listed in Table 1.

**Table 1:** Characteristics of the included randomized controlled trials This table summarizes key features of the four RCTs included in the meta-analysis, including study design, sample size, patient demographics, intervention details, comparator procedures, outcomes assessed, and length of follow-up [9-11] ASA: American Society of Anesthesiologists; IF: internal fixation; HA: hemiarthroplasty; RCT: randomized controlled trial; HHS: hyperosmolar hyperglycemic state; EQ-5D: EuroQol-5 dimension; CPT: Current Procedural Terminology

Study	Study design/country	Mean age (years)	Male sex, n (%)	ASA class I or II, n (%)	Patients (n)	Intervention (IF)	Comparison (HA)	Outcomes	Follow-up months
Lu et al. [9]	RCT/China	85.9	20 (25.6)	51 (65.3)	78	41 patients underwent IF with three 6.5-mm cannulated screws	37 patients underwent HA with a bipolar cemented prosthesis (lateral approach)	Complications, HHS, EQ-5D, mortality	38
Dolatowski et al. [10]	RCT/Norway	83.1	62 (28.3)	83 (38.0)	219	111 patients underwent IF with 2 partially threaded cancellous screws (8 mm)	108 patients underwent HA with cemented or cementless stem and modular head (lateral/posterior)	Complications, HHS, EQ-5D, mortality, reoperation	24
Wei et al. [11]	RCT/China	82.3	28 (27.1)	68 (66.0)	103	51 patients underwent IF with 3 cannulated screws	52 patients underwent HA with a bipolar uncemented prosthesis (lateral approach)	Complications, HHS, EQ-5D, mortality, reoperation	36
Parker and Cawley [12]	RCT/UK	81.8	17 (31.5)	30 (55.6)	54	27 patients underwent IF with Targon hip screws	27 patients underwent HA (cemented unipolar HA with CPT stem)	Mobility, pain, complications, mortality, blood loss, operative time, and independence	36
Total (4 RCTs)	-	83.3	127 (28.0)	232 (51.1)	454	230 patients underwent IF	224 patients underwent HA	Complications, function, mortality, and reoperation	24-38

Characteristics of the Included Studies

The four RCTs included in this review were published between 2017 and 2025 and conducted in China (n = 2), Norway (n = 1), and the United Kingdom (n = 1). Together, these trials enrolled 454 patients with nondisplaced femoral neck fractures, comprising 230 treated with IF and 224 with HA. The mean participant age ranged from 81.8 to 85.9 years. Across the pooled cohort, 124 patients (27.4%) were men, and 232 (51.3%) were classified as American Society of Anesthesiologists class I or II [9-11]. Follow-up duration was 24-36 months in the earlier studies, while the most recent trial (2025) extended follow-up to 36 months (Table 1).

Data Collection and Management

A standardized data-extraction spreadsheet was created in Microsoft Excel (Microsoft Corporation, Redmond, WA), based on the Cochrane data collection form for intervention reviews [17]. All tools (classification, scoring system, and scales) were free to use. The spreadsheet underwent pilot testing to ensure consistency and reliability. Two independent reviewers, MX and EJ, extracted data from the included studies, and any disagreements were resolved through discussion. The extracted data included study characteristics (author, year, country, study design, and sample size), patient characteristics (mean age, sex distribution, and relevant clinical background), intervention details (type of IF device, arthroplasty approach, use of cement, and perioperative protocols), as well as all predefined primary and secondary outcomes (Table 2).

**Table 2:** Complications and outcomes across included randomized controlled trials This table presents the distribution of postoperative complications and outcomes reported in the four RCTs comparing IF with HA for nondisplaced femoral neck fractures in elderly patients [9-11] IF: internal fixation; HA: hemiarthroplasty; NA: not available; NR: not reported; RCTs: randomized controlled trials

Complications/outcomes, n (%)	Lu et al. [9] (IF = 41/HA = 37)	Dolatowski et al. [10] (IF = 111/HA = 108)	Wei et al. [11] (IF = 51/HA = 52)	Parker and Cawley [12] (IF = 27/HA = 27)
Nonunion/fixation failure	5 (12.1)/0 (0.0)	17 (15.3)/1 (0.9)	5 (9.8)/0 (0.0)	2 (7.4)/0 (0.0)
Osteonecrosis	2 (4.8)/0 (0.0)	7 (6.3)/1 (0.9)	2 (3.9)/0 (0.0)	2 (7.4)/0 (0.0)
Peri-implant fracture	0 (0.0)/0 (0.0)	1 (0.9)/0 (0.0)	0 (0.0)/1 (1.9)	1 (3.7)/0 (0.0)
Prosthesis loosening	NA/1 (2.7)	NA/2 (1.8)	NA/0 (0.0)	NA/0 (0.0)
Deep infection	0 (0.0)/1 (2.7)	0 (0.0)/3 (2.7)	0 (0.0)/1 (1.9)	1 (3.7)/0 (0.0)
Pulmonary complications	NR	1 (0.9)/11 (10.1)	0 (0.0)/0 (0.0)	0 (0.0)/0 (0.0)
Other/not specified	8 (19.5)/8 (21.6)	NR	NR	NR
Total complications	16 (39.0)/10 (27.0)	27 (24.3)/20 (18.5)	7 (13.7)/3 (5.7)	4 (14.8)/0 (0.0)
Revision to arthroplasty	7 (17.0)/0 (0.0)	19 (17.1)/3 (2.7)	6 (11.7)/0 (0.0)	2 (7.4)/0 (0.0)
Revision fixation	0 (0.0)/NA	3 (2.7)/NA	0 (0.0)/NA	0 (0.0)/NA
Removal of screws	1 (2.4)/NA	5 (4.5)/NA	0 (0.0)/NA	0 (0.0)/NA
Debridement	0 (0.0)/1 (2.7)	0 (0.0)/0 (0.0)	0 (0.0)/1 (1.9)	0 (0.0)/NA
Total reoperations	8 (19.5)/2 (5.4)	27 (24.3)/8 (7.4)	6 (11.7)/2 (3.8)	2 (7.4)/0 (0.0)

Data Synthesis

A meta-analysis was conducted for outcomes reported by at least two studies. Mean differences (MDs) were used to assess continuous variables, while odds ratios (ORs) or risk ratios (RRs) were used for dichotomous outcomes, depending on the reporting format of the included studies. A random-effects model was used a priori for statistical analysis to account for expected clinical and methodological heterogeneity. All tools (classification, scoring systems, and scales) were free to use.

Statistical analysis was performed using Review Manager 5.4 (RevMan) software (The Cochrane Collaboration, London, UK) [18]. For dichotomous outcomes, ORs or RRs with 95% confidence intervals (CIs) were calculated, while continuous outcomes were analyzed using MDs with 95% CIs. For studies with multiple intervention arms, only relevant comparison groups were included to avoid double-counting of participants. Due to the limited number of included studies, formal sensitivity or meta-regression analyses were not performed.

Results were presented as forest plots with 95% confidence intervals (CIs) [18]. Heterogeneity among studies was evaluated using Cochrane’s Q test (χ²) and the I² statistic [18]. The I² statistic was interpreted as follows: 0%-40% may not indicate important heterogeneity, 30%-60% may represent moderate heterogeneity, 50%-90 % may indicate substantial heterogeneity, and 75%-100% may suggest considerable heterogeneity [18].

Risk of Bias and Quality Assessment

The methodological quality of RCTs was assessed using the Cochrane Risk of Bias 2.0 tool (Cochrane, London, UK) [19]. Risk-of-bias visualizations were generated using the robvis tool (University of Bristol, Bristol) [20]. Two reviewers assessed quality independently, with disagreements resolved by consensus.

Results

Study Selection

**Figure 1:**
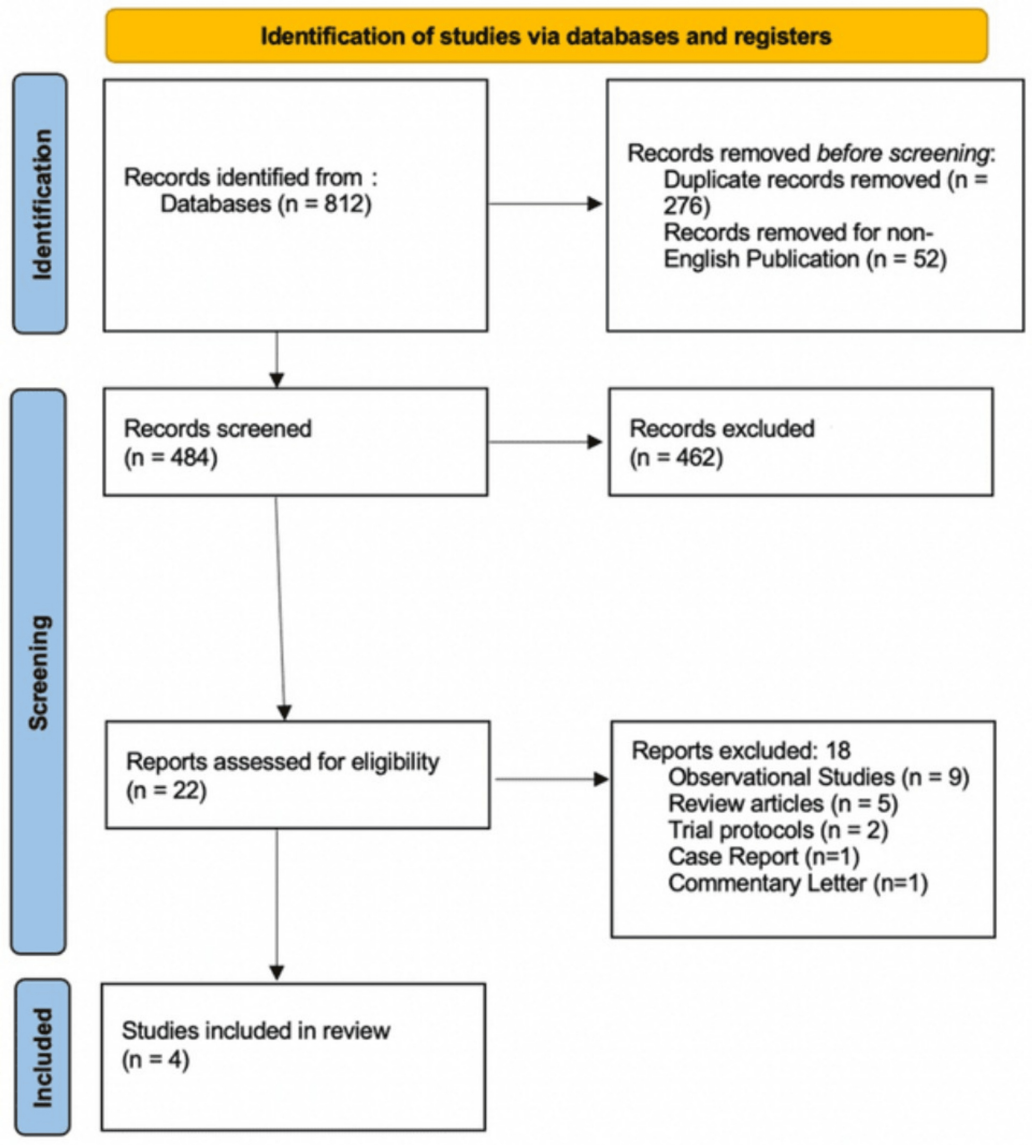
PRISMA 2020 flow diagram of study selection This flow diagram illustrates the study identification, screening, eligibility, and inclusion process, constructed using the PRISMA 2020 flow diagram tool [8] and following PRISMA 2020 guidelines [7] PRISMA: Preferred Reporting Items for Systematic Reviews and Meta-Analyses

The updated database search identified 812 records. After the removal of 276 duplicates and exclusion of 52 non-English publications, 484 unique studies were screened by title and abstract. Of these, 466 were excluded as irrelevant. The full texts of the remaining 18 articles were assessed for eligibility. Following review, 14 studies were excluded: seven observational studies, three review articles, two trial protocols, one case report, and one commentary letter. These studies were excluded due to nonrandomized design, lack of relevant IF vs. HA comparison, inclusion of displaced fractures, or insufficient outcome data. Ultimately, four RCTs met the inclusion criteria and were included in the meta-analysis (Figure 1).

Primary Outcomes

**Figure 2:**
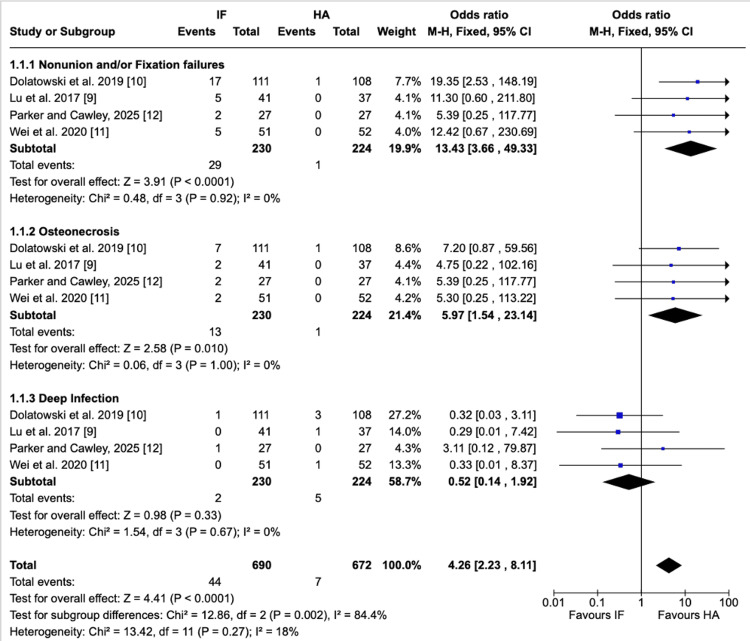
Implant-related complications Forest plot presenting the pooled analysis of implant-related complications comparing IF and HA for nondisplaced femoral neck fractures in four studies. Subgroup analyses are shown for nonunion/fixation failure, osteonecrosis, and deep infection Source: This image was generated using the RevMan software [17] IF: internal fixation; HA: hemiarthroplasty; M-H: Mantel-Haenszel; CI: confidence interval

Implant-related complications: Implant-related complications were significantly more common in the IF group compared with HA. For nonunion/fixation failure, rates were 12.6% in IF and 0.4% in HA, demonstrating a markedly higher risk with IF (OR = 13.43 (3.66-49.33), P < 0.0001; I² = 0%). For osteonecrosis, the incidence was 5.7% in IF vs. 0.4% in HA, also significantly favoring HA (OR = 5.97 (1.54-23.14), P = 0.01; I² = 0%) with no true nonunion events observed in the HA group. Any apparent events in the HA group likely reflect reporting or classification of prosthesis-related complications rather than true nonunion. For deep infection, no significant difference was observed between IF (0.9%) and HA (2.2%) (OR = 0.52 (0.14-1.92), P = 0.33; I² = 0%). When pooled, the overall rate of implant-related complications was substantially higher in IF than HA (19.1% vs. 3.1%) (OR = 4.26 (2.23-8.11), P < 0.0001; I² = 18%). Subgroup analysis confirmed significant differences between complication types (P = 0.002), with nonunion/fixation failure driving the excess risk in IF (Figure 2).

**Figure 3:**
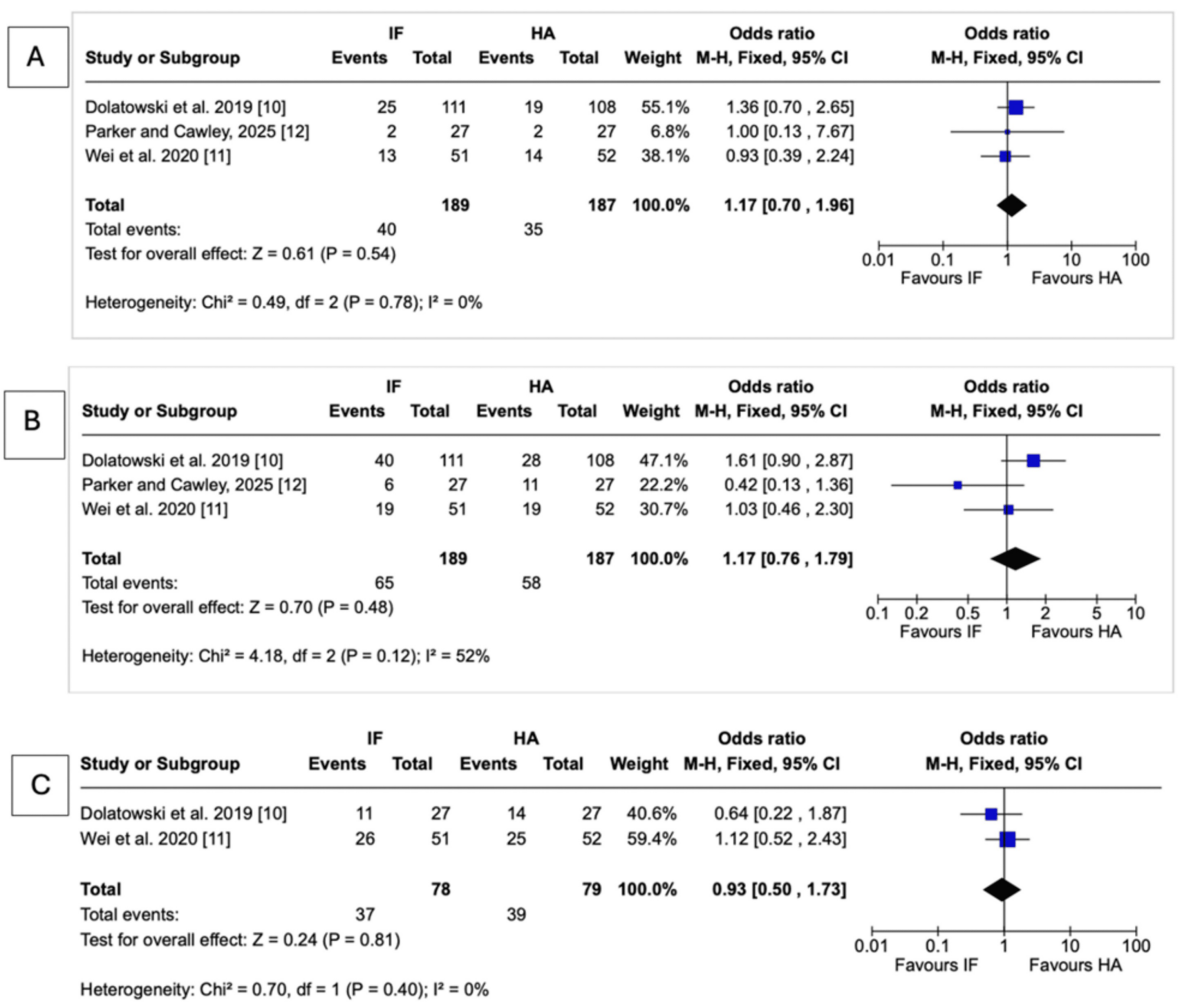
Mortality Forest plot presenting the pooled analysis of mortality comparing IF and HA for nondisplaced femoral neck fractures at (A) 12, (B) 24, and (C) 36 months in three studies Source: This image was generated using the RevMan software [17] IF: internal fixation; HA: hemiarthroplasty; M-H: Mantel-Haenszel; CI: confidence interval

Mortality: Mortality did not significantly differ between IF and HA at any follow-up point. At three months, mortality was 12.3% in the IF group compared with 9.3% in the HA group (OR = 1.29, 95% CI = 0.65-2.59; P = 0.46; I² = 14%). At 12 months, mortality rates were 21.2% for IF and 18.7% for HA (OR = 1.17, 95% CI = 0.70-1.96; P = 0.54; I² = 0%). At 24 months, pooled mortality was 34.4% in the IF group vs. 31.0% in the HA group (OR = 1.17, 95% CI = 0.76-1.79; P = 0.48; I² = 52%). At 36 months, data from two studies showed no significant difference, with mortality of 39.3% for IF and 39.7% for HA (OR = 0.93, 95% CI = 0.50-1.73; P = 0.81; I² = 0%) (Figure 3).

Secondary Outcomes

**Figure 4:**
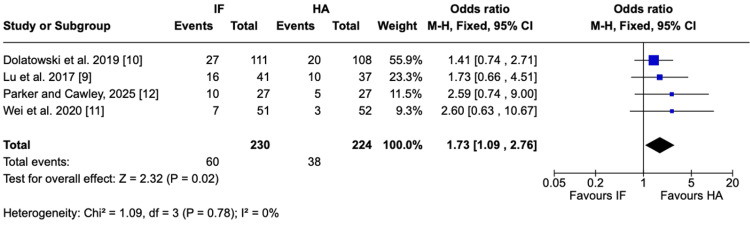
Total complications Forest plot presenting the pooled analysis of total complications comparing IF and HA for nondisplaced femoral neck fractures in four studies Source: This image was generated using the RevMan software [17] IF: internal fixation; HA: hemiarthroplasty; M-H: Mantel-Haenszel; CI: confidence interval

Total complications: Total complications were significantly higher in the IF group than in the HA group. Across four RCTs, complication rates were 26.1% for IF and 17.0% for HA, corresponding to a significantly increased odds of complications with IF (OR = 1.73, 95% = CI 1.09-2.76; P = 0.02; I² = 0%) (Figure 4).

Perioperative Outcomes

**Figure 5:**
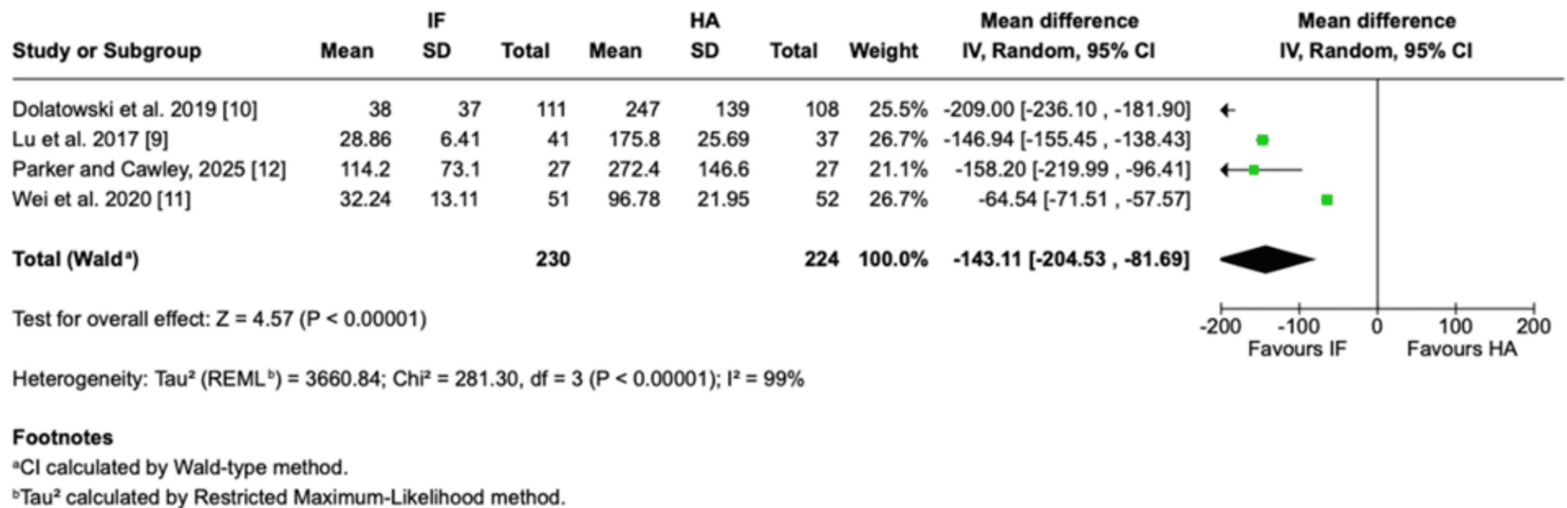
Perioperative blood loss Forest plot presenting the perioperative blood loss comparing IF and HA for nondisplaced femoral neck fractures in four studies Source: This image was generated using the RevMan software [17] IF: internal fixation; HA: hemiarthroplasty; SD: standard deviation; CI: confidence interval; REML: restricted maximum likelihood

Blood loss: Intraoperative blood loss was significantly lower in the IF group compared with the HA group. Across four RCTs, the MD was -143.11 mL (95% CI = -204.53 to -81.69; P < 0.00001), although heterogeneity was substantial (I² = 99%) (Figure 5).

**Figure 6:**
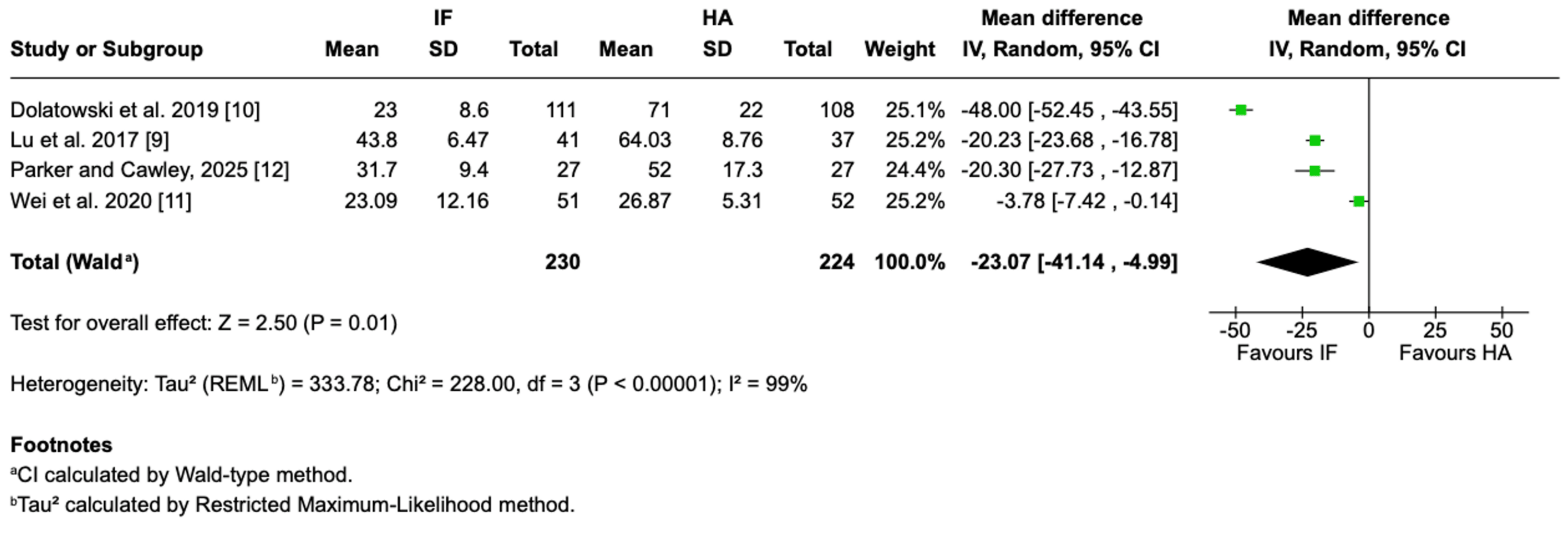
Operating time Forest plot presenting the pooled mean difference in operative time comparing IF and HA for nondisplaced femoral neck fractures in four studies Source: This image was generated using the RevMan software [17] IF: internal fixation; HA: hemiarthroplasty; SD: standard deviation; CI: confidence interval; REML: restricted maximum likelihood

Operative time: Operative time was also significantly shorter with IF than HA. The pooled MD was -23.07 minutes (95% CI = -41.14 to -4.99; P = 0.01), again with high heterogeneity (I² = 99%) (Figure 6).

**Figure 7:**
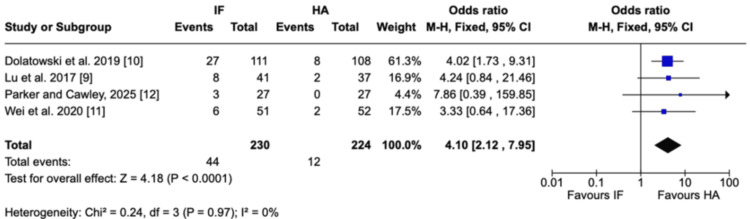
Reoperation Forest plot presenting the pooled odds ratio for reoperation comparing IF and HA for nondisplaced femoral neck fractures in four studies Source: This image was generated using the RevMan software [17] IF: internal fixation; HA: hemiarthroplasty; M-H: Mantel-Haenszel; CI: confidence interval; REML: restricted maximum likelihood

Risk of reoperations: The risk of reoperation was significantly higher in the IF than in the HA. Across four RCTs, reoperation rates were 19.1% for IF and 5.4% for HA, corresponding to more than a fourfold increased risk with IF (OR = 4.10, 95% CI = 2.12-7.95; P < 0.0001; I² = 0%) (Figure 7).

**Figure 8:**
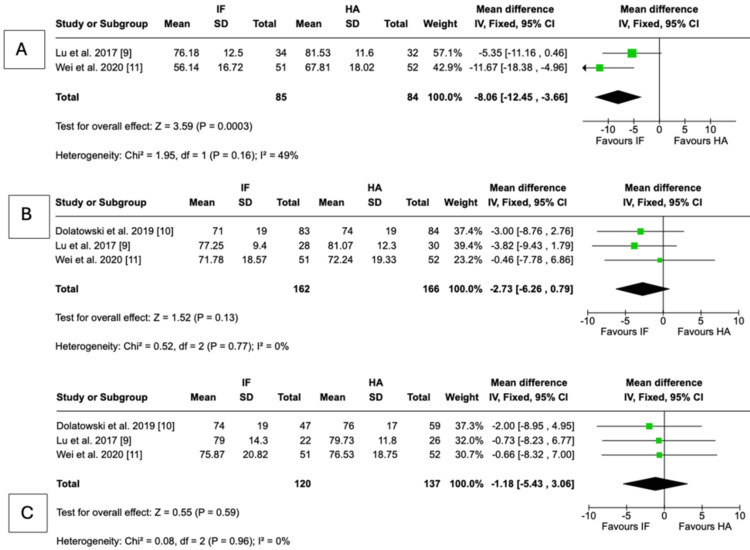
Harris Hip Score Forest plot presenting the pooled analysis of Harris Hip Score [13] comparing IF and HA for nondisplaced femoral neck fractures at (A) 6, (B) 12, and (C) 24 months in four studies Source: This image was generated using the RevMan software [17] IF: internal fixation; HA: hemiarthroplasty; CI: confidence interval; SD: standard deviation

Hip function: At six months, patients treated with HA demonstrated significantly better hip function than those treated with IF. The pooled MD in Harris Hip Score was -8.06 points (95% CI = -12.45 to -3.66; P = 0.0003; I² = 49%), favoring HA. At 12 months, HA continued to show a functional advantage, with mean Harris Hip scores higher than IF. The pooled MD was -2.73 points (95% CI = -6.26 to 0.79; P = 0.13; I² = 0%), although this did not reach statistical significance. At 24 months, no meaningful difference was observed between IF and HA. The pooled MD was -1.18 points (95% CI = -5.43 to 3.06; P = 0.59; I² = 0%) (Figure 8).

Methodological Quality and Risk of Bias Assessment

**Figure 9:**
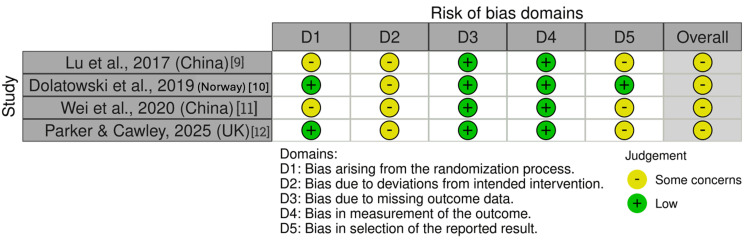
Risk of bias assessment of included randomized controlled trials The risk of bias was assessed using the Cochrane Risk of Bias 2 tool [19] across the domains of randomization process, deviations from intended intervention, missing outcome data, measurement of the outcome, and selection of the reported result, with visualization generated using the robvis tool [20]

The quality of the randomized studies was assessed using the Cochrane Collaboration tool. This is summarized in Figure 9. Overall, the RCTs showed some concerns in their risk of bias assessment [20].

Discussion/data analyses

The optimal management of nondisplaced FNFs in older adults remains controversial. IF has traditionally been favored due to its shorter operative time, reduced blood loss, and technical simplicity, but it carries well-documented risks of nonunion, fixation failure, and osteonecrosis that frequently necessitate reoperation [21,22]. HA, by contrast, eliminates the biological challenge of fracture healing at the expense of greater surgical invasiveness. Our updated meta-analysis, incorporating four RCTs including the recently published study by Parker and Cawley [12], provides the most contemporary synthesis of evidence on this subject.

Consistent with prior reviews [23,24], IF was associated with substantially higher implant-related complications compared with HA. Nonunion/fixation failure and osteonecrosis were the dominant contributors to this excess risk, while deep infection rates were comparable between the two procedures. The overall implant-related complication rate was more than fourfold higher with IF, and total complications and reoperations were similarly increased. The number needed to harm was approximately seven IF procedures, meaning that for every seven patients managed with IF, one additional implant failure or reoperation occurred compared with HA. These findings emphasize the mechanical and biological limitations of IF in the elderly, osteoporotic population.

Functional outcomes further support the superiority of HA, at least in the early postoperative period. HA was associated with significantly better Harris Hip Scores at six months and a trend toward improved scores at one year, though differences diminished by two years. These findings suggest that HA may accelerate early recovery of function and independence, an important advantage in frail patients, while long-term functional outcomes eventually converge. The updated 2025 trial [12] reinforced this pattern by demonstrating reduced pain and improved independence with HA at 9-24 months, though no significant differences were seen at three years.

Perioperative outcomes favored IF, with significantly lower blood loss and shorter operative time compared with HA. However, these differences, approximately 140-160 mL less blood loss and 20 minutes shorter operative duration, are unlikely to offset the burden of reoperations and mechanical failures associated with IF. Importantly, these perioperative disadvantages of HA did not translate into higher mortality, which remained comparable between IF and HA at all time points up to three years. Mortality across both groups was driven predominantly by age and comorbidities, rather than surgical technique [25].

Substantial heterogeneity was observed in perioperative outcomes, particularly for blood loss and operative time (I² = 99%). This likely reflects differences in surgical techniques, implant selection (e.g., cemented vs. uncemented HA), fixation methods, and perioperative protocols across studies conducted in different healthcare settings. Given the small number of included studies, formal subgroup or meta-regression analyses were not feasible. However, sensitivity analyses using a leave-one-out approach demonstrated consistent effect direction, suggesting that the overall findings are robust despite heterogeneity. A random-effects model was therefore used to account for between-study variability.

Compared with the previous meta-analysis of three RCTs [26], our study benefits from the inclusion of the trial by Parker and Cawley, which standardized interventions (cemented unipolar HA vs. Targon IF) and provided detailed baseline reporting. This reduced heterogeneity and strengthened the validity of the pooled estimates, directly addressing the previous limitations listed in the meta-analysis. While the overall conclusions-favoring HA for reduced complications and reoperations-remain consistent with earlier work, our updated analysis offers new granularity in subgroup outcomes and reinforces the robustness of these findings with longer-term follow-up.

Nonetheless, limitations remain. The total number of RCTs is still small, and sample sizes are modest, limiting the ability to perform stratified analyses. Key prognostic factors such as posterior tilt angle, preinjury function, and frailty status were not consistently reported, precluding subgroup evaluation [27,28]. Current UK guidance (National Institute for Health and Care Excellence CG124, updated 2023) supports IF for undisplaced intracapsular FNFs, while recognizing the importance of patient selection. Our findings are consistent with this, suggesting that although IF remains suitable for many patients, HA may be beneficial in selected higher risk individuals. In particular, fracture characteristics such as increased posterior tilt may increase the risk of fixation failure. In these cases, primary arthroplasty may be a more appropriate option, even in fractures classified as Garden I-II. Overall, these findings support an individualized approach to management, taking into account patient frailty, functional status, and fracture characteristics, in line with multidisciplinary orthogeriatric practice.

Moreover, blinding of participants and assessors was not feasible, introducing potential detection bias. High statistical heterogeneity was observed in perioperative outcomes, likely reflecting differences in surgical expertise, rehabilitation pathways, and health system contexts across countries. Furthermore, health-economic data remain absent, yet are critical for informing practice, given the higher implant cost of HA but reduced reoperation rates compared with IF [29].

## Conclusions

Taken together, this updated meta-analysis provides compelling evidence that HA was superior to IF for the management of nondisplaced FNFs in older adults, reducing implant-related complications and reoperations while maintaining comparable survival. IF retains advantages of shorter surgery and less blood loss, but these benefits appear to be outweighed by the risk of failure and need for revision surgery. Future research should focus on large pragmatic multicenter trials or registry-based studies to provide real-world generalizability, alongside cost-effectiveness analyses and patient-reported outcomes to guide decision-making. Particular attention should be paid to identifying subgroups, such as patients with minimal posterior tilt, better bone quality, or lower anesthetic risk, who may still benefit from IF.

## Appendices

Supplementary table search strategy

Database: MEDLINE (via PubMed)

(("Femoral Neck Fractures"[Mesh] OR "Femoral Neck Fracture"[tiab] OR "Hip Fractures"[Mesh] OR "hip fracture*"[tiab] OR "femoral neck fracture*"[tiab] OR "intracapsular fracture*"[tiab] OR "subcapital fracture*"[tiab] OR "undisplaced fracture*"[tiab] OR "nondisplaced fracture*"[tiab]) AND ("Arthroplasty, Replacement, Hip"[Mesh] OR "Hemiarthroplasty"[tiab] OR "Hip Arthroplasty"[tiab] OR "Endoprosthesis"[tiab] OR "Hip Prosthesis"[tiab]) AND ("Internal Fixators"[Mesh] OR "Fracture Fixation, Internal"[Mesh] OR "internal fixation"[tiab] OR "screw fixation"[tiab] OR "cannulated screw*"[tiab] OR "targon"[tiab] OR "dynamic hip screw"[tiab]) AND ("Randomized Controlled Trial"[Publication Type] OR "randomized"[tiab] OR "randomised"[tiab] OR "RCT"[tiab] OR "clinical trial"[tiab]))

Database: EMBASE

('femoral neck fracture'/exp OR 'hip fracture'/exp OR (hip NEXT/1 fracture*):ab,ti OR (femoral NEXT/1 neck NEXT/1 fracture*):ab,ti OR intracapsular:ab,ti OR subcapital:ab,ti OR nondisplaced:ab,ti OR undisplaced:ab,ti) AND ('hip arthroplasty'/exp OR hemiarthroplast*:ab,ti OR 'hip prosthesis'/exp OR endoprosthes*:ab,ti) AND ('internal fixation device'/exp OR 'fracture fixation'/exp OR 'internal fixation':ab,ti OR screw*:ab,ti OR 'cannulated screw*':ab,ti OR 'dynamic hip screw':ab,ti OR targon:ab,ti) AND ('randomized controlled trial'/exp OR random*:ab,ti OR rct:ab,ti OR 'clinical trial*':ab,ti)

Database: Cochrane CENTRAL

(femoral neck fracture* OR hip fracture* OR intracapsular fracture* OR subcapital fracture* OR nondisplaced fracture* OR undisplaced fracture*) AND (hemiarthroplast* OR hip arthroplast* OR endoprosthes* OR hip prosthesis) AND (internal fixation OR screw fixation OR cannulated screw* OR dynamic hip screw OR targon) AND (random* OR RCT OR "clinical trial")
